# Deficiency of diacylglycerol Kinase ζ promotes Beclin1-mediated autophagy *via* the mTOR/TFEB signaling pathway: Relevance to maladaptive cardiac hypertrophy

**DOI:** 10.7150/ijms.88134

**Published:** 2024-01-01

**Authors:** Yumei Liu, Han Zhang, Yaxian Lin, Peipei Qian, Yuan Yin, Ganglin Zou, Jinxin Zhang, Haining Zhang

**Affiliations:** 1 Key Laboratory of Molecular Target & Clinical Pharmacology and the State & NMPA Key Laboratory of Respiratory Disease, School of Pharmaceutical Sciences & the Fifth Affiliated Hospital, Guangzhou Medical University, Guangzhou, 511436, P.R. China.; 2 Department of Pharmacology, Jiaying University, Meizhou, 514000, P.R. China.; 3 Department of Stomatology, the First Affiliated Hospital, Sun Yat-sen University, Guangzhou 510080, China; 4 Xiamen Cardiovascular Hospital, Xiamen University, Xiamen, 361005, P.R. China.; 5 Affiliated Guangxi International Zhuang Medical Hospital, Guangxi University of Traditional Chinese Medicine, Guangxi, 530021, P.R. China.; 6 Nanhai Mental Health Center, People's Hospital of Nanhai District, Foshan, 528200, P.R. China.; 7 Department of Medical Statistics, School of Public Health, Sun Yat-sen University, Guangzhou, 510080, China

**Keywords:** Autophagy, DGKζ, TFEB, Cardiac hypertrophy

## Abstract

The activation Gq protein-coupled receptors (GPCRs) is a crucial factor contributing to maladaptive cardiac hypertrophy, and dysregulation of autophagy is implicated in its prohypertrophic effects. Previous studies have shown that diacylglycerol kinase zeta (DGKζ) can suppress cardiac hypertrophy by inhibiting the diacylglycerol (DAG)-PKC pathway in response to mechanical strain or growth agonists such as endothelin-1 (ET-1). However, the involvement of DGKζ in autophagy regulation remains poorly understood. In this study, we aimed to investigate the role of DGKζ in autophagy regulation during maladaptive cardiac hypertrophy. We found that Beclin1-mediated autophagy was involved in the development of maladaptive cardiac hypertrophy and dysfunction in response to prohypertrophic challenges of transverse aortic constriction (TAC) or ET-1. Deficiency of DGKζ promoted Beclin1-mediated autophagy, aggravated adverse cardiac remodeling, and cardiac dysfunction, which could be ameliorated by genetic deletion of Beclin1 or TFEB. Mechanistically, the deficiency of DGKζ disrupted the activation of AKT/mTOR signaling, the association between mTOR and TFEB, and favored the nuclear translocation of TFEB from the cytoplasm, leading to enhanced activation of Beclin1-mediated autophagy through ULK1/Beclin1 signaling and TFEB-dependent Beclin1 transcription. Taken together, these results suggest that the mechanisms by which DGKζ alleviates pathological cardiac hypertrophy may involve the regulation of Beclin1-mediated autophagy through the mTOR/TFEB signaling pathway.

## Introduction

Cardiac hypertrophy is the response of heart to various biomechanical and pathophysiological stimuli such as aging, myocardial ischemia and hypertension, which characterized by an increased cardiomyocyte size, protein synthesis as well as re-expression of fetal genes. Initially, the impaired cardiac functions provoked by those stimuli could be compensated by cardiac hypertrophy. However, the progressive cardiac hypertrophy eventually resulted in decompensated cardiac dysfunction and cardiac failure 1. Therefore, the development of effective therapeutic interventions for cardiac hypertrophy is crucial for controlling and treating heart failure.

The Gq protein-coupled receptor (GPCR) pathway is widely recognized to play a crucial role in the development of cardiac hypertrophy 2. Endothelin (ET)-1, a potent vasoconstrictive peptide primarily produced by the endothelium, exerts a broad range of biological effects by primarily acting on GqPCR. Numerous studies have indicated that increased production and activity of ET-1 in the vasculature contribute to hypertension, cardiac hypertrophy, and heart failure. The activation of GqPCR promotes cardiac hypertrophy by stimulating phospholipase C-mediated hydrolysis of phosphatidylinositol 1,4,5-bisphosphate (PIP2) to produce 1,4,5-triphosphate (IP3) and DAG. The diacylglycerol kinase (DGK), as a DAG kinase, controls cell DAG levels by phosphorylating DAG to phosphatidic acid (PA), thereby terminating an arm of Gq protein signaling 3. We and others have demonstrated that DGKζ, the primary DGK isoform in the heart, can prevent cardiac hypertrophy induced by ET-1 and other GqPCR agonists such as pressure overload and angiotensin Ⅱ by inhibiting the DAG-PKC pathway 4, 5. However, the exact mechanism by which DGKζ regulates cardiac hypertrophy remains to be determined.

Autophagy is a highly conserved process that facilitates the bulk degradation and recycling of cytoplasmic components, including long-lived proteins and organelles, in a lysosome-dependent manner, which plays a crucial role in maintaining metabolic and cellular homeostasis 6. However, autophagy dysregulation is implicated in various pathological processes, such as cancer, neurodegenerative diseases, and cardiovascular disorders 7-9. Emerging evidence suggests that sustained activation of GqPCR pathways is related to dysregulation of autophagy, which may contribute to its prohypertrophic effects 9-12. Nevertheless, the role of DGKζ in autophagy regulation during cardiac hypertrophy remains poorly understood.

In this study, we provided evidence that a reduction in DGKζ levels in hypertrophic hearts enhances Beclin1-dependent autophagy, leading to worsened cardiac hypertrophy and dysfunction. The regulation of Beclin1-dependent autophagy by DGKζ may play a role in its impact on cardiac hypertrophy through the modulation of the mTOR/TFEB signaling pathway.

## Materials and methods

### Animals and experimental protocols

All the animal studies reported in this article were performed in accordance with *the Guide for the Care and Use of Laboratory Animals*, Eighth Edition, (2011, published by The National Academies Press). The experimental procedures were approved by *Institutional Animal Care and Use Committee of Guangzhou Medical University.*

C57BL/6 male mice (22-25 g body weight) were obtained from the experimental animal center of Guangdong Province (Guangzhou, China) and received a standard diet and water *ad libitum*. Cardiac hypertrophy was induced by permanent Transverse Aortic Constriction (TAC). Briefly, mice were anesthetized by sodium pentobarbital (50 mg/kg, ip) and artificially ventilated with an animal ventilator (DH-140, Zhejiang, China). After thoracotomy at the second intercostal space, the transverse aortic arch was ligated between the innominate artery and the left common carotid artery by the 7-0 silk suture against a 26-gauge needle. Mice underwent the same surgical procedure without TAC served as sham control. Thoracic Aorta Color Doppler ultrasound was performed 1 to 3 days after surgery to confirm the ligation of transverse aorta.

A total of 48 mice were randomized into 6 groups: (i) sham group; (ii) TAC group; (iii) Scramble+TAC group; (Ⅳ) Beclin1 shRNA+TAC group; (Ⅴ) DGKζ shRNA+TAC group; (Ⅵ) (DGKζ+Beclin1) shRNA+TAC group. Mice in Scramble+TAC group, Beclin1 shRNA+TAC group, DGKζ shRNA+TAC group and (DGKζ+Beclin1) shRNA+TAC group received intramyocardial injections of lentivirus containing scramble shRNA, Beclin1 shRNA and/or DGKζ shRNA one week before TAC.

### *In vivo* cardiac-specific gene manipulation

The method for delivering genes specifically to the heart *in vivo* was carried out according to previous protocols 13, 14. Briefly, the mouse was anesthetized and the heart was quickly exposed through a left thoracotomy at the fifth intercostal space. A total of 30 μL Lentivirus solution containing scramble shRNA, Beclin1 shRNA and/or DGKζ shRNA (~2×10^7^ PFU) was injected into the myocardium of the left ventricle in three sites (ventral, dorsal, and lateral wall of the left ventricle). Transverse Aortic Constriction was performed one week after the virus injection. Mice were sacrificed, and the hearts were harvested and processed for histologic analysis or western blotting analysis 8 weeks after TAC.

### Echocardiography

Echocardiography was performed to evaluate cardiac function by using a high-resolution imaging system (Vevo 2100, VisualSonics Inc., Ontario, Canada) equipped with a 25 MHz imaging transducer. Briefly, two-dimensional echocardiographic views of parasternal long-axis and short-axis as well as the apical four chamber were obtained. Cardiac systolic function parameters including left ventricular anterior wall in systole (LVAWs), left ventricle ejection fraction (LVEF), left ventricle fractional shortening (LVFS) and cardiac diastolic function parameters such as the ratio of E-wave velocity to A-wave velocity (E/A), isovolumetric relaxation time (IVRT), isovolumetric constriction time (IVCT) and the early diastolic mitral annulus velocity/late diastolic mitral annulus velocity (e'/a') were analyzed according to the instruction of the Vevo 2100.

### Histologic analysis

The hearts were fixed in 4% paraformaldehyde, embedded by paraffin and sectioned (5 μm thickness). After routine dewaxing, the sections were stained with hematoxylin-eosin (H&E) and Masson trichrome and imaged with a microscope (Nikon Instruments Inc, Japan). By using Image J software (NIH, version 1.30, http://rsb.info.nih.gov/ij/), 2-dimensional cross-sectional areas of cardiomyocyte were evaluated, and the fibrosis in left ventricle (LV) was determined by the area of fibrotic tissue (blue=collagen) over LV area (above background). More than five fields in three different sections were examined for each mouse by the researcher who was blinded to the treatments.

### Electron microscopy

Heart tissues were cut into sections of 1 mm^3^ and pre-fixed in 2.5% glutaraldehyde. After washed, the sections were incubated with 1% OsO4 at 4℃ for 3h, followed by dehydrated in graded series of ethanol, and flat embedded in epoxy resin. Ultrathin sections were counterstained with uranyl acetate and lead citrate and observed under a transmission electron microscope (HITACHI H-600, Japan).

### Cell isolation, culture and treatment

Sprague-Dawley pups (1-2-day-old) were purchased from the experimental animal center of Guangdong Province (Guangzhou, China). The animals were handled according to National Institute of Health Guidelines on the Care and Use of Experimental Animals. Primary cardiomyocytes were prepared as described previously 4. Briefly, ventricles were obtained from pups, and cardiomyocytes were isolated by digestion with trypsin. Cells were cultured in Dulbecco's modified Eagles's medium (DMEM) supplemented with 10% Fetal Bovine Serum (FBS), BrdU (10^-4^ mol/L), penicillin (100 U⁄mL) and streptomycin (100 µg⁄mL). After 24 hours of serum deprivation, cardiomyocytes were treated with ET-1 (10^-7^ mol/L) (Sigma-Aldrich, St. Louis, MO, USA) for 24 h to induce myocardial hypertrophy.

### Quantitation of autophagy with HBAD-mRFP-GFP-LC3 adenovirus

After being infected with tandem mRFP-GFP-LC3 adenoviral particles at a MOI of 50 (Hanbio Biotechnology Co., Ltd. Shanghai, China) for 24 hours, the cells were treated with ET-1 for the indicated time. Fluorescent signals excited at 488 nm (green) and 560 nm (red) were obtained using a confocal laser scanning microscopy (Nikon America Inc., Melville, NY). After merged, red puncta and yellow puncta were manually counted to determine the number of autolysosomes and the number of autophagosomes, respectively. The quantification of autolysosomes and autophagosomes was performed by a researcher who was blinded to the treatments. A total of thirty randomly selected cells per experimental group were analyzed.

### Immunostaining of cardiomyocytes

The cells were fixed with 4% paraformaldehyde and permeabilized with 0.1% TritonX-100 after treatment. After blocking with PBS containing 2% fat-free milk, the cells were immunostained by using primary antibody against TFEB (1:200, Cat#13372-1-AP, Proteintech Group, Inc. Rosemont, IL, USA) and the second antibody conjugated to Alex-488 (green) (1:1000, Santa Cruz, CA, USA). F-actin of myocytes was probed using phallodin conjugated to TRITC (red) (Sigma-Aldrich, St. Louis, MO, USA) and nuclei were co-stained with DAPI (blue) (Invitrogen, Grand Island, NY). Fluorescent signals were obtained with a fluorescence microscope (Olympus 1×2-UCB-2).

### Cell size measurement

F-actin of cardiomyocytes was immunostained using phallodin conjugated to TRITC. Myocyte size was assessed by immunofluorescent microscopy and measured by Image J software. Thirty randomly selected cells per experimental group were analyzed.

### Diacylglycerol (DAG) assay

Cell lysates were prepared in RIPA buffer and clarified by centrifugation. DAG levels were measured using enzyme-linked immunosorbent assay (ELISA) kits (Cloud-Clone Corp, Houston, TX) following the manufacturer's instructions and DAG concentration in each sample was calculated based on the standard curve by a microplate reader (BioTek Synergy2; BioTek, Winooski, VT, USA).

### Generation of TFEB shRNA construct

shRNA against TFEB or Beclin1 was constructed into pLKO.1 lentiviral vector (Open Biosystems, Ottawa, Canada) following the manufacturer's instruction. The construct was verified by DNA sequence analysis.

### Lentiviral preparation and infection

Lentivirus expressing shRNAs against DGKζ, Beclin1 or TFEB were prepared by co-transfecting DGKζ shRNA lentiviral plasmids (TRCN0000025394, TRCN0000025395, TRCN0000025398) (Open Biosystems, Ottawa, Canada), Beclin1 shRNA or TFEB shRNA lentiviral plasmids with packaging plasmid into HEK-293T cells using FuGENE6 reagent (Roche, Indianapolis, IN, USA). Empty lentiviral plasmid (pLKO.1) and lentiviral plasmid containing scramble sequences served as vector control and non-silencing control, respectively. Cardiomyocytes were infected by adding 50 MOI lentivirus particles to the medium for 24 hours, and then cultured in the newly replaced medium for another 24 hours before serum deprivation and ET-1 treatment. Specificity and degree of knockdown were confirmed by western blotting 72 h after virus infection.

### Co-immunoprecipitation (IP) and western blotting analysis

Cell lysates were prepared in RIPA buffer and TFEB precipitates were generated from cardiomyocyte protein extracts with anti-TFEB antibody and protein A/G beads (Santa Cruz, CA, USA). Immunoprecipitates and the proteins extracted from heart tissues or cardiomyocytes were separated by SDS-PAGE and transferred to PVDF membranes (Roche Molecular Biochemicals, Mannheim, Germany). The membranes were blocked and detected with anti-LC3 antibody (Cat#3868), anti-Beclin-1 antibody (Cat#3738), anti-phosphorylated-Beclin1 antibody (Cat#13825), anti-p62 antibody (Cat#5114), anti-phosphorylated-Akt antibody (Cat#9271), anti-Akt antibody (Cat#9272), anti-phosphorylated-AMPK antibody (Cat#2531), anti-AMPK antibody (Cat#2532), anti-phosphorylated-mTOR antibody (Cat#2971), anti-mTOR antibody (Cat#2972), Phospho-ULK1Ser757 antibody (#14202), ULK1 antibody (#8054) from Cell Signaling Technology (Beverly, MA), anti-DGKζ antibody (Cat#SC-8722, Santa Cruz, CA, USA) or anti-β-actin antibody (Cat#BS6007M, Bioworld Technology, St. Louis Park, MN, USA), respectively. The density of target bands was accurately determined by the computer-aided Quantity One analysis system. To avoid variability from different treatment time, mice or the proteins for detection *per se*, the expression of proteins in the heart of TAC mice was normalized to that of the sham mice in each time point, and β-actin served as a loading control.

### mRNA isolation and Quantitative Real-time PCR

Total RNA was extracted by TRIzol reagent (TaKaRa Biomedical Technology, Beijing, Co., Ltd), and equal amount of purified RNA was reversely transcribed using the RNA PCR Kit according to the manufacturer's instructions. The cDNAs were amplified and real-time PCR was performed with primers specific for BNP, β-MHC and Beclin1 (Life technology, Invitrogen, Ltd. Paisley PA4 9RF, UK). The mRNA levels of BNP, β-MHC and Beclin1 were normalized to that of β-actin.

### Chromatin immunoprecipitation (ChIP) assay

ChIP was conducted using the SimpleChIP Enzymatic Chromatin IP Kit (Cell Signaling Technology, 9003) as previously described 4. After treatment, cells were collected and cross-linked with 1% formaldehyde for 10 minutes, quenched with glycine, and then sonicated using a disruptor to generate DNA fragments ranging from 200 to 1000 base pairs. Immunoprecipitation was carried out using an anti-TFEB antibody (Cell Signaling Technology, 83010), while a normal Rabbit IgG (Cell Signaling Technology, 2729) was used as a control. Following reversal of protein-DNA cross-linking, the DNA was purified and used for quantitative PCR with primers flanking TFEB binding site at the promoter of Beclin1 (-1420 to -1290: forward 5ʹ-AGGACCAGTGAATGAGAACAGAC-3ʹ, reverse 5ʹ-ACCATCAACGCCATGTGACT-3ʹ; -540 to -340: forward 5ʹ- GTGACTTGCTCCTTAGGGGA -3ʹ, reverse 5ʹ- TATACATGGCGTGCTGTGCT -3ʹ; -880 to -670: forward 5ʹ-ATCAAGTCCCTGCCCACTTG-3ʹ, reverse 5ʹ- TCTTGCCTTTCTCCACGTCC -3ʹ).

### Beclin1 luciferase assay

Beclin1 1.5 kb promoter sequence containing putative a TFEB-binding site was cloned into pLV-Pro-luci reporter plasmids (pLV-TFEB, inovogen Tech. Co, Chongqing, China). Following lentivirus preparation, myocytes were coinfected with lentiviruses expressing a Beclin1-luciferase reporter and/or DGKζ shRNA, TFEB shRNA. The cells were cultured in DMEM/10% FBS for 24 hours and then subjected to serum deprivation for another 24 hours. After treatment with ET-1, luciferase activity was measured from lysates using a Luciferase Reporter Assay System (Promega).

### Statistical analysis

All data were expressed as mean ± standard deviation and analyzed by software of GraphPad Prism 5.0 (GraphPad Software Inc). Student *t* test was used to evaluate the differences between two groups, while one-way ANOVA was used for more than two groups followed by Tukey post-hoc test. A value of *P*< 0.05 was considered as statistically significant.

## Results

### Beclin1-mediated autophagy was upregulated during cardiac hypertrophy in response to TAC *in vivo* and ET-1 *in vitro*

**Figure 1:**
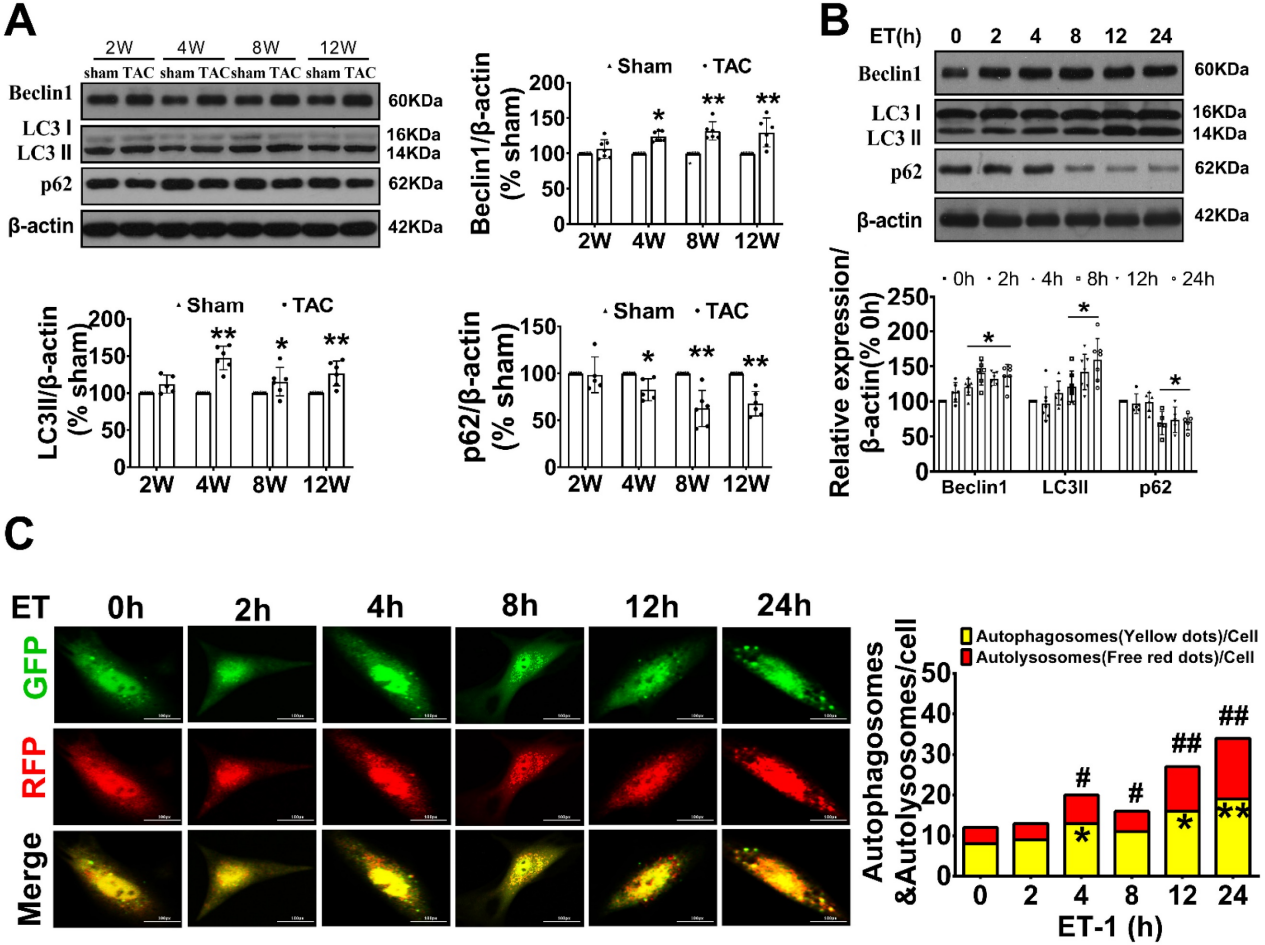
** Beclin1-mediated autophagy was upregulated with cardiac hypertrophy* in vivo* and *in vitro*.** (A) The expression of LC3, beclin1 and p62 in hypertrophic heart were determined by western blotting at indicated time point after TAC (Student *t* test, *n*=5-6, *P< 0.05 or **P< 0.01 *vs.* sham. The expression of proteins in TAC mice was normalized to that of the sham mice in each time point, and β-actin served as a loading control). (B) Cardiomyocytes were challenged by ET-1 and the expression of LC3, beclin1 and p62 were examined by western blotting at indicated time point (one-way ANOVA, *n*=5-6, *P< 0.05 *vs.* 0h). (C) After infected with mRFP-GFP-LC3 adenoviral particles for 24h, the cells were challenged by ET-1. Fluorescent signals were captured with the confocal laser scanning microscopy at indicated time point and the number of autolysosomes and autophagosomes was determined by counting of red puncta or yellow puncta, respectively (one-way ANOVA, *P< 0.05 or **P< 0.01 *vs.* 0h in yellow puncta. ^#^*P*< 0.05, ^##^*P*< 0.01 *vs.* 0h in red puncta. Thirty randomly selected cells per experimental group were analyzed).

To investigate the potential involvement of autophagy in adverse cardiac hypertrophy, we initially assessed the level of autophagy in the hearts of TAC mice and in neonatal cardiomyocytes treated with ET-1. Compared to the sham group, the expression of LC3 II, a marker for autophagosomal vesicles, was elevated, while the expression of p62, a ubiquitin binding adaptor protein that serves as an indicator of autophagic degradation, was significantly reduced in the hearts of mice after TAC for 4 weeks. Consistently, treatment of cardiomyocytes with ET-1 (10^-7^ mol/L) for 8-24 hours also significantly increased the expression of LC3 II and the degradation of p62. Importantly, we observed a significant upregulation of Beclin1, a key initiator of autophagy required for autophagosome formation, in the hearts of TAC mice and in ET-1-treated cardiomyocytes. These results suggest that autophagy is actively upregulated by hypertrophic stimuli TAC and ET-1 (Figure 1A-B).

To confirm our observations, autophagic flux was further monitored using the tandem RFP-GFP-LC3 fluorescence analysis based on that the GFP fluorescence is quenched due to sensitive to the acidic and/or proteolytic conditions of the lysosome lumen, whereas mRFP fluorescence is not. Therefore, colocalization of GFP and mRFP fluorescence indicates an autophagosome that has not fused with a lysosome. In contrast, an mRFP signal without GFP corresponds to an autolysosome. We observed that ET-1 treatment time-dependently increased the numbers of yellow puncta and the numbers of red puncta, which indicated increased autophagosomes and autolysosomes, respectively (Figure 1C).

### Suppression of Beclin1-mediated autophagy ameliorated pathological cardiac remodeling and cardiac dysfunction

**Figure 2:**
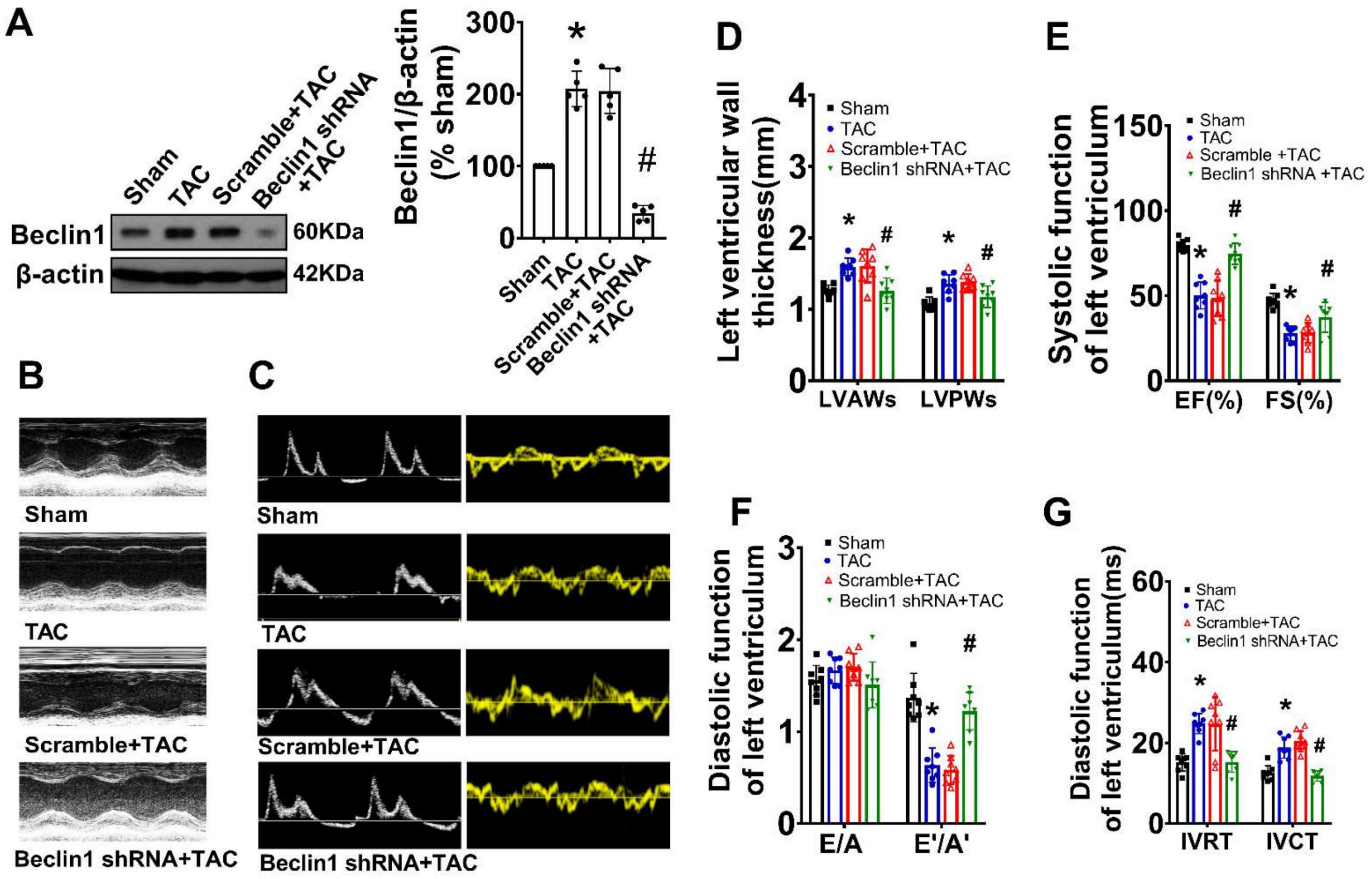
** Suppression of Beclin1-mediated autophagy ameliorated cardiac dysfunction.** Autophagy was perturbed by a cardiac-specific gene knockdown of Beclin1 through *in vivo* intramyocardial delivery of lentivirus-encoded Beclin1 shRNA one week prior to TAC. (A) Cardiac hypertrophy was induced by TAC in mice and the expression of Beclin1 in the heart was determined by western blotting (one-way ANOVA, *n*=5, *P<0.05 *vs.* sham, *^#^P*<0.05* vs.* TAC). (B) Representative M-mode echocardiograms. (C) Representative transmitral flow and tissue doppler echocardiograms. (D-G) The analyzed results of cardiac function obtained from mice in each experimental group (one-way ANOVA, *n*=7-8, *P< 0.05 *vs.* sham, ^#^*P*<0.05 *vs.* TAC).

**Figure 3:**
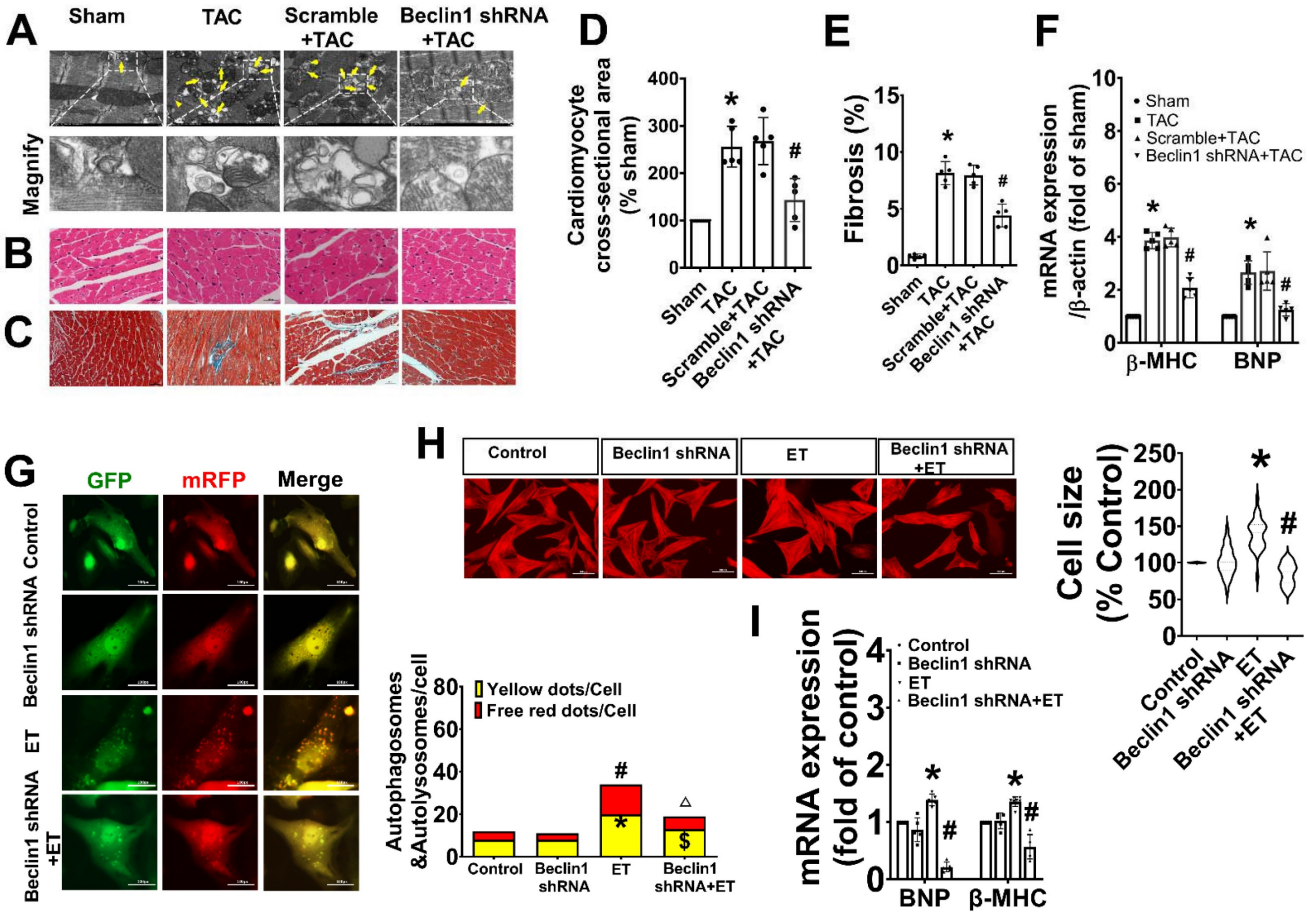
** Inhibition of Beclin1-mediated autophagy mitigated adverse cardiac remodeling and cardiac hypertrophy.** (A) Ultrastructure analysis showing the autophagosomes (multimembrane vacuoles, arrow heads) and autolysosomes (electron dense structures, arrows) in the myocardium 8 weeks after TAC. (B) Representative cross-sectional images with H&E staining. (C) Representative cross-sectional images showing interstitial fibrosis with Masson staining. (D) Analysis results for cardiomyocyte cross-sectional area (one-way ANOVA, *n*=5, *P< 0.05 *vs*. sham, ^#^*P*< 0.05 *vs.* TAC). (E) Analysis results for fibrosis (one-way ANOVA, *n*=5, *P< 0.05 *vs.* sham, ^#^*P*< 0.05 *vs.* TAC). (F) mRNA level of β-MHC and BNP in hypertrophic heart were determined by real time RCR from mice in each experimental group (one-way ANOVA, *n*=5, *P< 0.05 *vs.* sham and ^#^*P*< 0.05 *vs.* TAC, β-actin served as a loading control). (G) Neonatal cardiomyocytes pretreated with Beclin1 shRNA were subjected to ET-1 for 24h. Autolysosomes and autophagosomes were determined by use of a tandem mRFP-GFP-LC3 adenovirus in cardiomyocytes. The red puncta indicated autolysosomes and the yellow puncta indicated autophagosomes, respectively (one-way ANOVA, *P< 0.05 or ^#^*P*< 0.05 *vs.* Control in yellow puncta or red puncta, respectively. ^$^*P*< 0.05 *vs.* ET in yellow puncta. ^△^*P*< 0.05 *vs.* ET in red puncta. Thirty randomly selected cells per experimental group were analyzed). (H) The cell size was assessed by Image J (one-way ANOVA, *n*=30, *P < 0.05 *vs.* control, ^#^*P*< 0.05 *vs.* ET). (I) mRNA levels of β-MHC and BNP in cardiomyocytes were determined by real time RCR (one-way ANOVA, *n*=5, *P< 0.05 *vs.* control, ^#^*P*< 0.05 *vs.* ET-1, β-actin served as a loading control).

As Beclin1 plays a crucial role in the initiation of autophagy and biogenesis of autophagosomes, we sought to investigate the impact of Beclin1-mediated autophagy on cardiac remodeling and dysfunction following TAC by using a cardiac-specific gene knockdown of Beclin1 through *in vivo* intramyocardial delivery of lentivirus-encoded Beclin1 shRNA one week prior to TAC. Our results showed that compared to TAC mice, Beclin1 knockdown decreased the thickness of the left ventricular anterior wall (LVAWs) and left ventricular posterior wall (LVPWs) and improved cardiac dysfunction, as evidenced by increased LVEF, LVFS, and e'/a', as well as shortened IVRT and IVCT 8 weeks after TAC (Figure 2). Electron microscopic analysis revealed the presence of cytoplasmic autophagosomes vesicles in the hearts of sham mice (Figure 3A). In comparison to sham mice, more autophagosomes were found in the hearts of TAC mice and scramble shRNA-treated mice. Enlarged cardiomyocytes, deranged myocardial fibers, cardiac fibrosis between myocardial fibers, and increased mRNA expression of fetal gene BNP and β-MHC were also observed in the hearts of TAC mice by H&E staining, Masson-Trichrome staining, and real-time PCR, all of which were reduced by Beclin1 knockdown compared to TAC mice (Figure 3A-F).

Similarly, in cultured primary cardiomyocytes, knocking down of Beclin1 effectively reduced the numbers of autophagosomes and autophagolysosomes in response to ET-1 challenge (Figure 3G). Treatment of cardiomyocytes with ET-1 for 24 hours induced an increase in myocyte size and hypertrophic gene expression of BNP and β-MHC, all of which were suppressed by Beclin1 knockdown (Figure 3H-I). These findings suggest that Beclin1 knockdown decreased cardiac autophagy and improved long-term adverse cardiac remodeling induced by TAC.

### DGKζ was downregulated both in the heart of TAC mice and in the ET-1-treated cardiomyocytes

**Figure 4:**
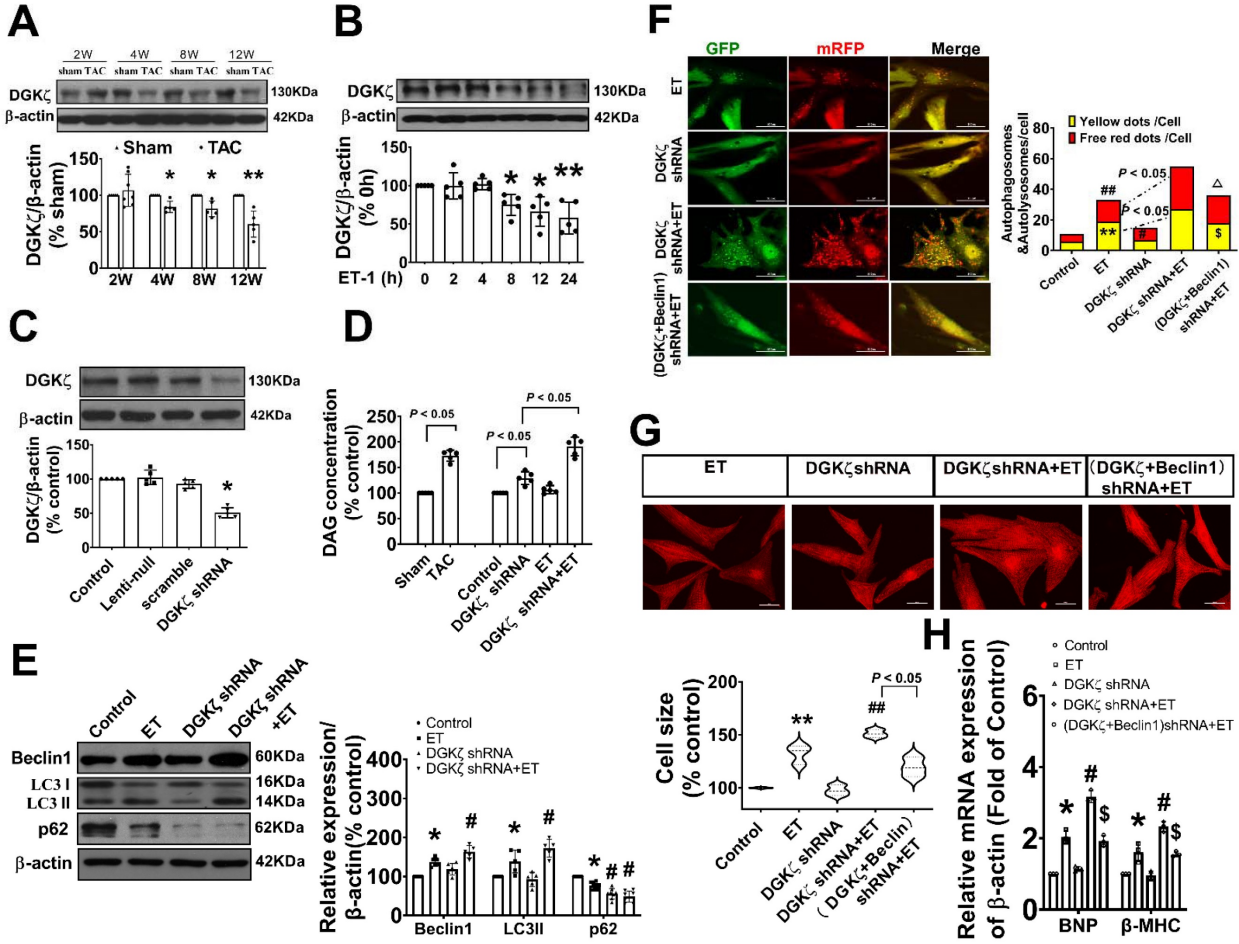
** DGKζ was decreased in response to the prohypertrophic stimuli *in vivo* and *in vitro,* and DGKζ deficiency linked to Beclin1-mediated autophagy during myocardial hypertrophy** (A) Cardiac hypertrophy was induced by TAC in mice and the expression of DGKζ in the heart was determined by western blotting at indicated time point after surgery (Student *t* test, *n*=5, *P< 0.05 or **P< 0.01 *vs.* sham. The expression of proteins in TAC mice was normalized to that of the sham mice in each time point, and β-actin served as a loading control). (B) The expression of DGKζ was determined by western blotting in ET-1-treated cardiomyocytes for indicated time point (one-way ANOVA, *n*=5, *P<0.05 or **P<0.01 *vs.* 0h). (C) The level of DGKζ was successfully downregulated in cardiomyocytes by infecting lentiviral DGKζ shRNA. Lentiviral plasmid containing no targeted sequences or scramble sequences served as vector control and non-silencing control (one-way ANOVA, *n*=5, *P<0.05 *vs.* control). (D) The level of DAG was measured by ELISA kits in heart tissue 12 weeks after TAC and in cardiomyocytes after ET-1 treatment for 24h (one-way ANOVA, *n*=5). (E) Cardiomyocytes were challenged by ET-1 and the expression of LC3, beclin1 and p62 were examined by western blotting (one-way ANOVA, *n*=5-6, *P< 0.05 *vs.* control, ^#^*P*< 0.05 *vs.* ET). (F) Autolysosomes and autophagosomes were determined by use of a tandem mRFP-GFP-LC3 adenovirus in cardiomyocytes. The red puncta indicated autolysosomes and the yellow puncta indicated autophagosomes, respectively (one-way ANOVA, **P< 0.01 *vs.* control in yellow puncta. ^#^*P*< 0.05 or ^##^*P*< 0.01 *vs.* control in red puncta. ^$^*P*< 0.05* vs.* DGKζ shRNA+ET in yellow puncta. ^△^*P*< 0.05 *vs.* DGKζ shRNA+ET in red puncta. Thirty randomly selected cells per experimental group were analyzed). (G) The cell size was assessed by Image J (one-way ANOVA, *n*=30, **P<0.01 *vs.* control, ^##^*P*<0.01 *vs.* ET). (H) mRNA level of β-MHC and BNP in cardiomyocytes were determined by real time RCR (one-way ANOVA, *n*=3, *P< 0.05 *vs.* control, ^#^*P*< 0.05 *vs.* ET, ^$^*P*< 0.05 vs. DGKζ shRNA+ET, β-actin served as a loading control).

To investigate the potential involvement of DGKζ in regulating autophagy, we first examined the level of DAG and the expression of DGKζ in the hearts of TAC mice and cardiomyocytes treated with ET-1. As shown in Figure 4A-D, both TAC and ET-1 treatment led to increased levels of DAG in the hearts of TAC mice and cardiomyocytes. The expression of DGKζ in the hearts of mice was slightly increased 2 weeks after TAC, but significantly decreased from weeks 4 to 12 compared to the sham mice. Consistent with the reduced levels of DGKζ in the hearts of TAC mice, ET-1 treatment also significantly decreased the expression of DGKζ protein in neonatal cardiomyocytes.

### Deficiency of DGKζ linked to the Beclin1-mediated autophagy during maladaptive cardiac hypertrophy

To investigate the potential impact of reduced DGKζ on autophagy, we evaluated autophagy levels in primary cardiomyocyte cultures exposed to ET-1 after DGKζ knockdown using lentiviral DGKζ shRNA. As shown in Figure 4C-D, targeted shRNA against DGKζ significantly reduced DGKζ expression and increased the DAG concentration in cultured cardiomyocytes, indicating that DGKζ critically regulated the cellular level of DAG. Compared to the ET group, DGKζ knockdown promoted ET-1-induced upregulation of Beclin1 expression, increased the accumulation of LC3-II, degradation of p62, and increased the numbers of autophagosomes and autophagolysosomes after ET-1 challenge. However, the increases in autophagosomes and autophagolysosomes due to DGKζ deficiency were hampered by knocking down Beclin1 (Figure 4E-F).

Additionally, DGKζ knockdown significantly increased myocardial cell size and the mRNA expression of fetal genes BNP and β-MHC induced by ET-1. Notably, the promoting effect of DGKζ knockdown on ET-1-induced increases of myocardial cell size and the mRNA expression of fetal genes BNP and β-MHC was markedly blunted by combined treatment with Beclin1 shRNA (Figure 4G-H), indicating that deficiency of DGKζ prompted cardiomyocyte hypertrophy by enhancing Beclin1-mediated autophagy.

**Figure 5:**
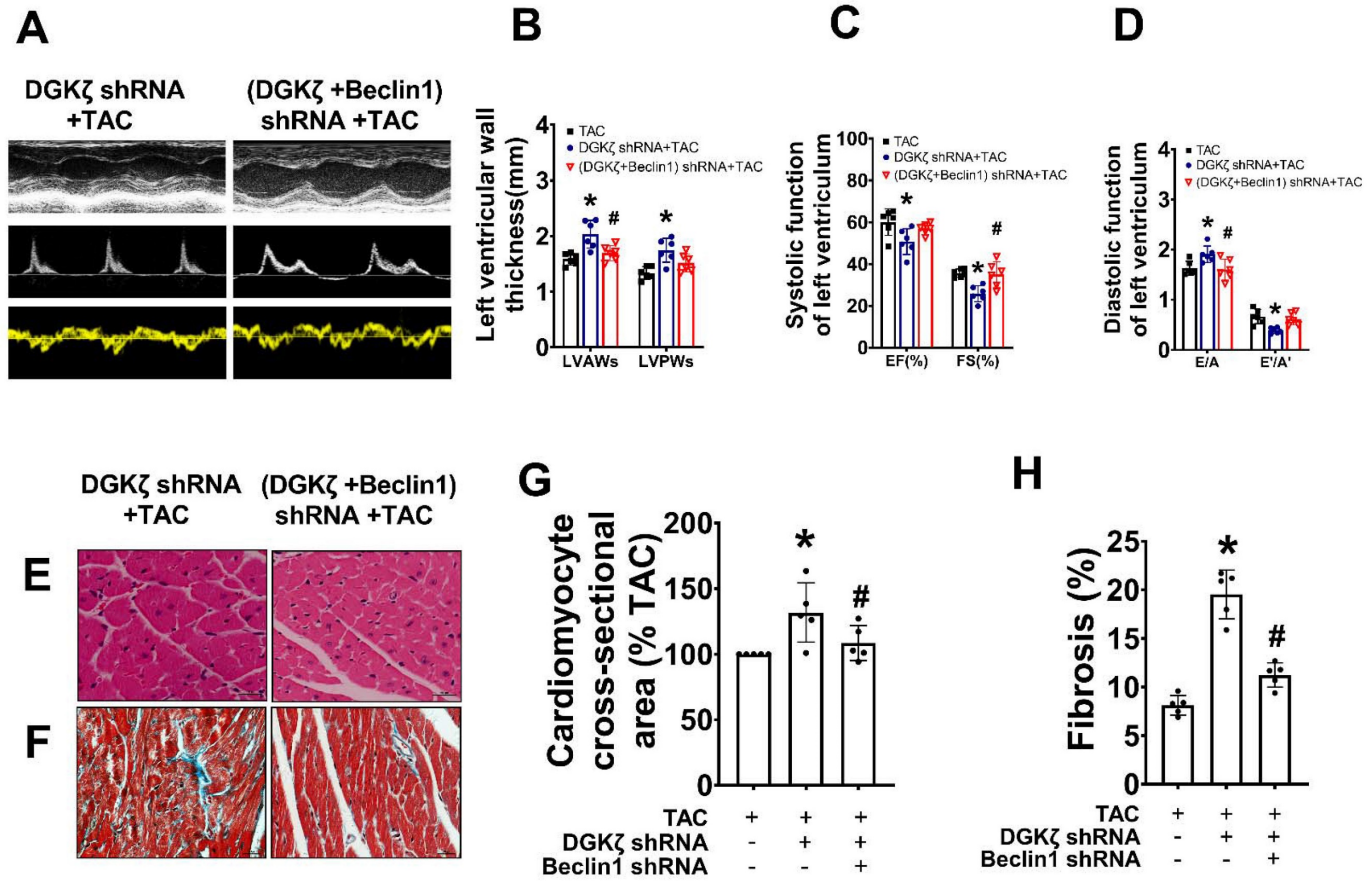
** Deficiency of DGKζ promoted cardiac dysfunction by augmenting Beclin1-mediated autophagy.** (A) Representative M-mode echocardiograms, transmitral flow and tissue doppler echocardiograms. (B) The analyzed results of LVAW and LVPW obtained from mice in each experimental group ((one-way ANOVA, *n*=6, *P<0.05 *vs.* TAC, ^#^*P*<0.05 *vs.* DGKζ shRNA+TAC). (C) The analyzed results of EF and FS obtained from mice in each experimental group ((one-way ANOVA, *n*=6, *P<0.05 *vs.* TAC, ^#^*P*<0.05 *vs.* DGKζ shRNA+TAC). (D) The analyzed results of E/A and e'/a' obtained from mice in each experimental group ((one-way ANOVA, *n*=6, *P<0.05 *vs.* TAC, ^#^*P*<0.05 *vs.* DGKζ shRNA+TAC). (E) Representative cross-sectional images with H&E staining. (F) Representative cross-sectional images with masson staining. (G) Analysis results for cardiomyocyte cross-sectional area (one-way ANOVA, *n*=5, *P< 0.05 *vs.* TAC, ^#^*P*<0.05 *vs.* DGKζ shRNA+TAC). (G) Analysis results for fibrosis (one-way ANOVA, *n*=5, *P< 0.05 *vs.* TAC, ^#^*P*< 0.05 *vs.* DGKζ shRNA+TAC).

We further investigated whether the deficiency of DGKζ affects cardiac remodeling and dysfunction by enhancing Beclin1-mediated autophagy. Specifically, we delivered lentivirus-encoded DGKζ shRNA and Beclin1 shRNA *via in vivo* intramyocardial injection one week prior to TAC. The DGKζ shRNA led to worsened systolic and diastolic dysfunction, as evidenced by a reduction in LVEF, LVFS, and e'/a', but an increase in the thickness of the left ventricular wall and the ratio of E/A 8 weeks after TAC. This indicates an accelerated progression of heart failure compared to TAC mice. Histological analysis revealed that mice with DGKζ deficiency had more deranged myocardial fibers, loss of continuity of the myofilaments, increased cardiomyocyte cross-sectional area, and cardiac fibrosis than TAC mice. Importantly, all of these effects were attenuated by combined treatment with Beclin1 shRNA (Figure 5A-H).

### DGKζ deficiency promoted the activation of Beclin1-mediated autophagy via an AKT/mTOR-dependent, but AMPK-independent pathway

**Figure 6:**
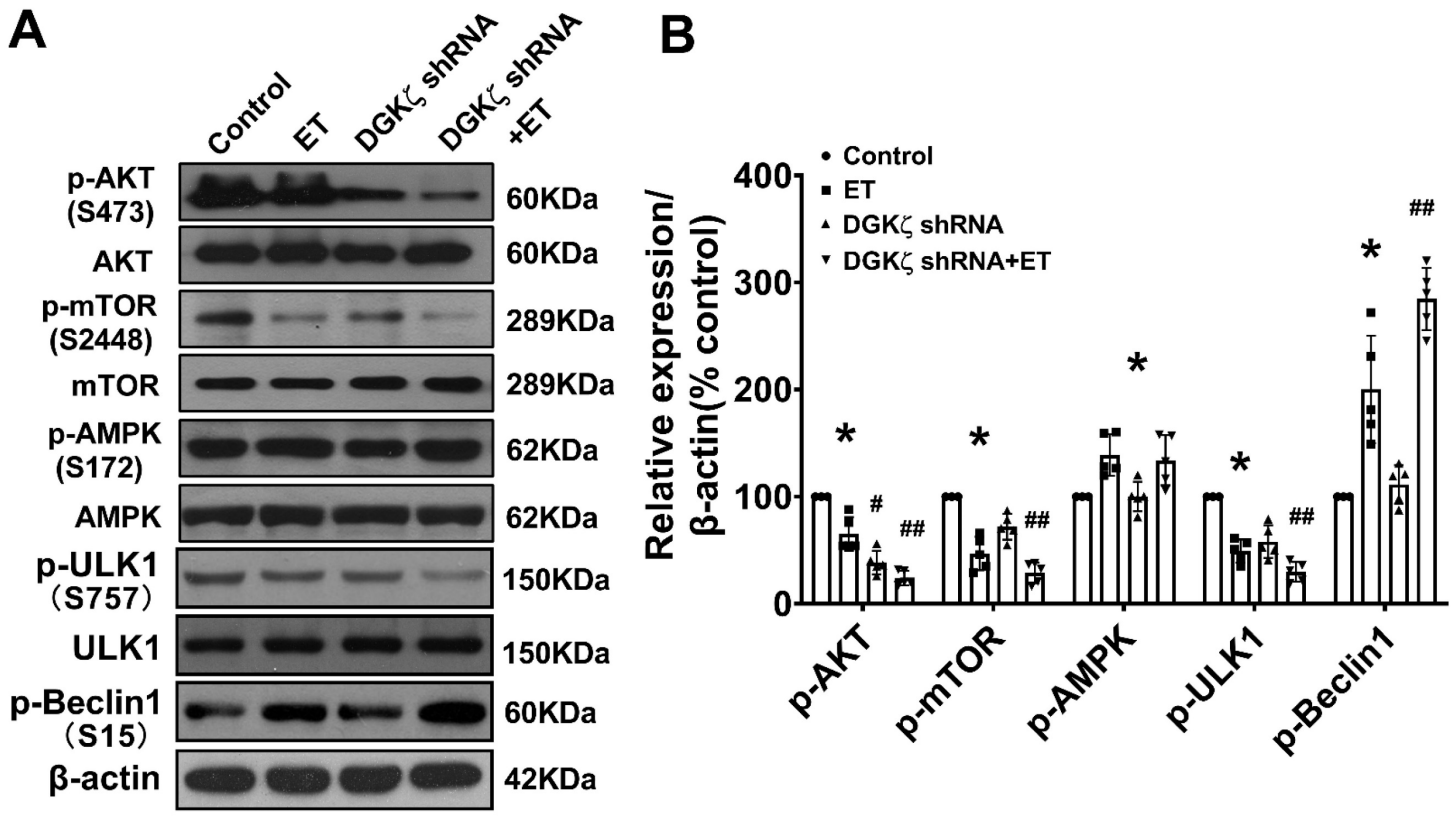
** AKT/mTOR dependent signaling is involved in the enhancement of cardiac autophagy by DGKζ deficiency** Cardiomyocytes infected with lentivirus expressing DGKζ shRNA were subjected to ET-1 for 24h. The total protein expression and phosphorylated level of AKT, mTOR, AMPK, ULK1 and Beclin1 were examined by western blotting, respectively. (A) Representative blots depicting total and phosphorylated proteins. (B) The analyzed results of p-AKT/AKT level, p-mTOR/mTOR level, p-AMPK/AMPK level, p-ULK1/ULK1 level and p-Beclin1 level (one-way ANOVA, *n*=5, *P< 0.05* vs.* control.*^#^P<* 0.05 or *^##^P<* 0.01 *vs.* ET).

To investigate the mechanisms underlying the enhancement of autophagy mediated by DGKζ deficiency, we examined the phosphorylation levels of AKT/mTOR and AMPK, which are classic pathways involved in the regulation of autophagy. As shown in Figure 6, ET-1 treatment decreased the phosphorylation levels of Akt and mTOR, but increased the phosphorylation of AMPK. After DGKζ knockdown, the phosphorylation of Akt and mTOR was further inhibited compared to that of the ET-1 group. However, the phosphorylation of AMPK induced by ET-1 was not affected by DGKζ knockdown. These results suggest that DGKζ deficiency enhances cardiomyocyte autophagy in an AKT/mTOR-dependent manner. The activation of mTOR can directly phosphorylate ULK1 at Ser757, leading to the inhibition of autophagy. Conversely, disrupting ULK1 phosphorylation by mTOR inhibition activates the VPS34-BECLIN1-VPS15 complex to initiate autophagy. After ET-1 challenge, the expression of ^Ser757^p-ULK1 was downregulated, while ^Ser15^p-Beclin1 was upregulated, and knocking down of DGKζ further enhanced the effects of ET-1 on the phosphorylation of ULK1 and Beclin1, confirming the activation of Beclin1-mediated autophagy by DGKζ deficiency.

### Deficiency of DGKζ impaired the interaction of mTOR with TFEB and promoted the nuclear translocation of TFEB

**Figure 7:**
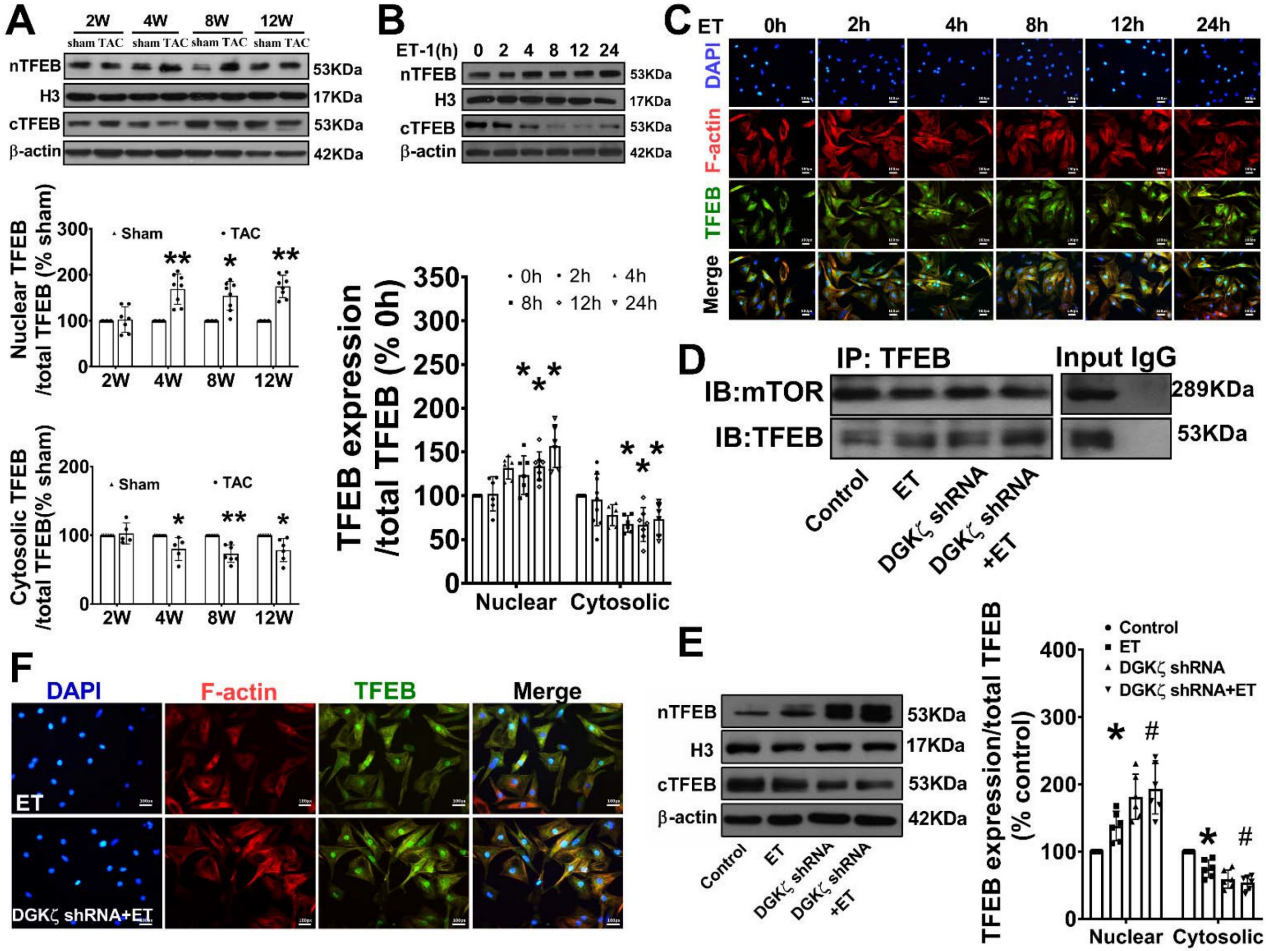
** Deficiency of DGKζ impaired the interaction of mTOR with TFEB and favored the nuclear translocation of TFEB.** (A) Representative blot and the analyzed results of the nuclear TFEB level and cytoplasm TFEB levels in heart tissues after TAC (Student *t* test, *n*=7-8, *P< 0.05 or **P< 0.01 *vs.* sham. The expression of proteins in TAC mice was normalized to that of the sham mice in each time point, and β-actin served as a loading control). (B) Representative blot and the analyzed results of the nuclear TFEB level and cytoplasm TFEB levels in ET-1-treated cardiomyocytes (one-way ANOVA, *n*=6, *P<0.05 *vs.* 0h). (C) Immunofluorescent staining showing the translocation of TFEB from cytoplasm to nucleus induced by ET-1. (D) The representative image of immunoprecipitation assay showing that mTOR interacted with TFEB in cardiomyocytes. Input was used as a positive control and IgG was used as a negative control. (E) Representative blot and the analyzed results of the nuclear TFEB level and cytoplasm TFEB levels in cardiomyocytes after DGKζ knockdown (one-way ANOVA, *n*=5-6, *P<0.05 *vs.* control, *^#^P<*0.05 *vs.* ET). (F) Immunofluorescent staining showing the translocation of TFEB from cytoplasm to nucleus induced by ET-1 after DGKζ knockdown.

As mTOR also regulates autophagy at the transcriptional level by modulating localization and activity of transcription factor EB (TFEB), a master regulator of the transcriptional activation of genes of lysosomal and autophagy, we tested whether interaction of mTOR with TFEB and subsequent nuclear translocation of TFEB are involved in the regulation of Beclin1-mediated autophagy by DGKζ. We found that the level of TFEB in the cytoplasm was significantly reduced but increased in the nucleus in TAC mice heart from 4W after TAC compared to the sham mice. Similarly, treatment of cardiomyocytes with ET-1 decreased the cytoplasmic level of TFEB in a time-dependent manner, while induced TFEB translocation from the cytoplasm to the nucleus and increased the nuclear accumulation of TFEB (Figure 7A-C). Importantly, ET-1 treatment decreased mTOR binding to TFEB, and DGKζ knockdown further inhibited the interaction of mTOR with TFEB induced by ET-1, promoted TFEB nuclear translocation and nuclear accumulation of TFEB (Figure 7D-F).

### TFEB contributed to the upregulated Beclin1 expression mediated by deficiency of DGKζ

**Figure 8:**
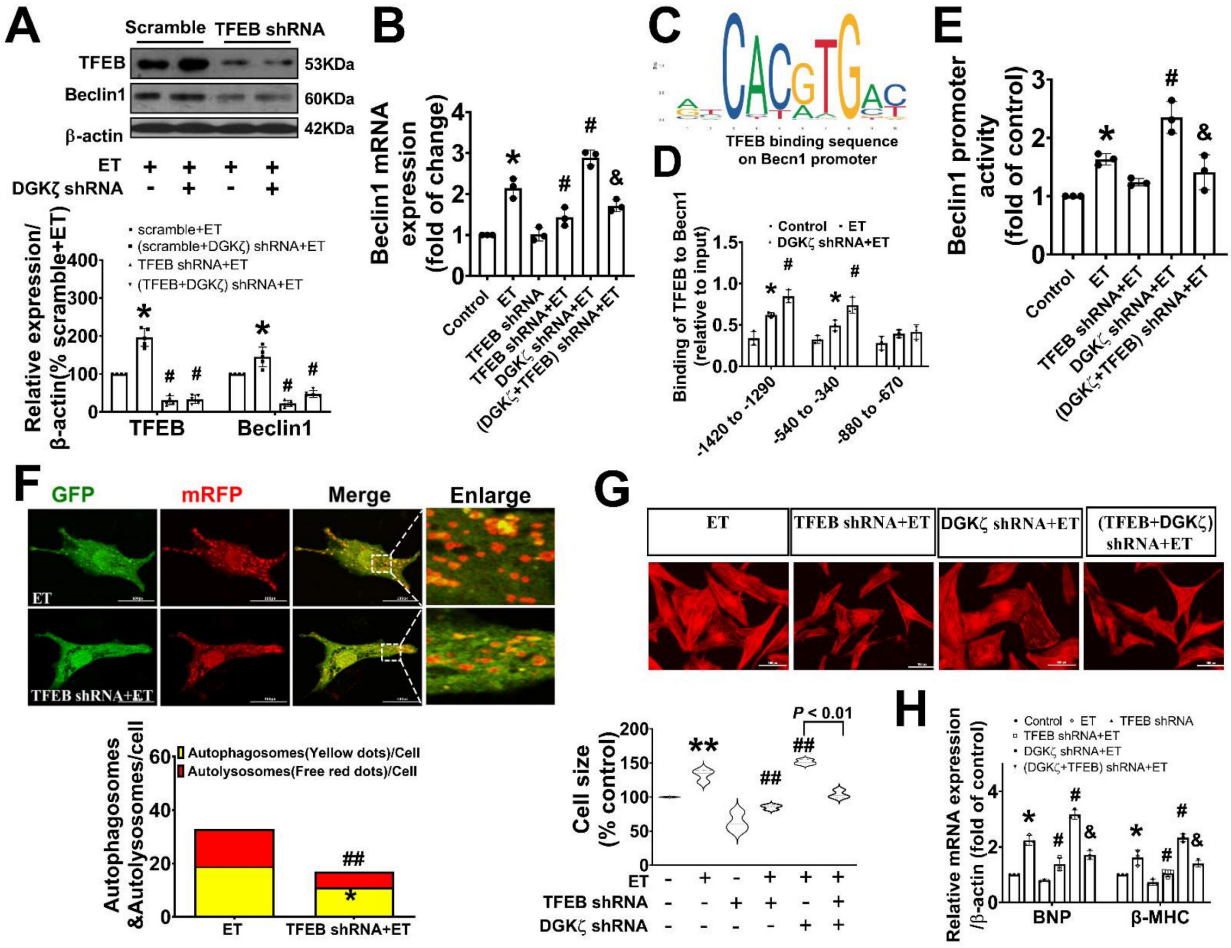
** Genetic knockdown of TFEB abrogates Beclin1-mediated autophagy and ameliorate ET-1-induced myocardial hypertrophy.** (A) Representative blot and the analyzed results of the expression of TFEB and Beclin1 in ET-1-treated cardiomyocytes (one-way ANOVA, *n*=5, *P<0.05* vs.* scramble+ET, ^#^*P*<0.05 *vs.* (scramble+DGKζ) shRNA+ET). (B) mRNA levels of Beclin1 in cardiomyocytes were determined by real time RCR (one-way ANOVA, *n*=3, *P< 0.05 *vs.* control, ^#^*P*< 0.05 vs. ET, ^&^*P*< 0.05 *vs.* DGKζ shRNA+ET, β-actin served as a loading control). (C) Schematic representation of conserved binding sequences identified in the promoter regions of Becn1 using JASPAR. (D) Occupancy of Becn1 promoter regions -1420 to -1290, 540 to -340, and -880 to -670 by TFEB, as determined by ChIP assay (one-way ANOVA,* n*=3, *P< 0.05 *vs.* control, ^#^*P*<0.05 *vs.* ET, β-actin served as a loading control). (E) Luciferase activity in cardiomyocytes transfected with the Becn1 luciferase reporter constructs (one-way ANOVA, *n*=3, *P<0.05 *vs.* control, ^#^*P*<0.05 vs. ET, ^&^*P*<0.05 *vs.* DGKζ shRNA+ET). (F) Autolysosomes and autophagosomes were determined by use of a tandem mRFP-GFP-LC3 adenovirus in cardiomyocytes. The red puncta indicated autolysosomes and the yellow puncta indicated autophagosomes, respectively (Student *t* test, *P< 0.05 *vs.* ET in yellow puncta. ^##^*P*< 0.01 *vs.* ET in red puncta. Thirty randomly selected cells per experimental group were analyzed). (G) The cell size was assessed by Image J (one-way ANOVA, *n*=30, **P < 0.01 *vs.* control, ^##^*P*< 0.01 *vs.* ET). (H) mRNA level of β-MHC and BNP in cardiomyocytes were determined by real time RCR (one-way ANOVA, *n*=3, *P< 0.05 *vs.* control, ^#^*P*<0.05 *vs.* ET, ^&^*P*<0.05 *vs.* DGKζ shRNA+ET, β-actin served as a loading control).

Given our findings that deficiency of DGKζ increased the level of Beclin1 protein induced by ET-1, we hypothesized that the upregulation of Beclin1 expression may be due to transcriptional activation of TFEB mediated by deficiency of DGKζ. Real-time PCR showed that deficiency of DGKζ significantly increased the mRNA levels of Beclin1 induced by ET-1. However, when TFEB was silenced, both the increases in Beclin1 mRNA and protein were abolished, indicating a crucial role for TFEB in the regulation of Beclin1 expression (Figure 8A-B).

TFEB regulates target genes by binding to the coordinated lysosomal expression and regulation (CLEAR) motifs. Through an in silico search, we identified three potential binding sites (-1420 to -1290, 540 to -340, and -880 to -670) within the promoter of the Beclin1 gene. A ChIP analysis revealed that ET-1 treatment significantly increased the occupancy of TFEB within the -1420 to -1290 and 540 to -340 regions of the Beclin1 promoter, but not in the other predicted site. These occupancies were further enhanced upon DGKζ silence (Figure 8C-D). To determine whether the expression of Beclin1 was modulated by TFEB through Beclin1 promoter, we generated Beclin1 reporter constructs and performed dual luciferase reporter assays. ET-1 treatment strongly induced luciferase reporter activity, which was further enhanced by DGKζ silence. However, knocking down of TFEB abolished the promotional effect mediated by ET-1 and DGKζ deficiency, suggesting that transcriptional activity of TFEB may actively contribute to the increased expression of Beclin1 mediated by deficiency of DGKζ (Figure 8E).

### Genetic knockdown of TFEB mitigated the autophagy and myocardial hypertrophy elicited by DGKζ deficiency

To confirm the involvement of TFEB in the enhancement of autophagy and myocardial hypertrophy mediated by DGKζ deficiency, cardiomyocytes were infected with lentiviral TFEB shRNA. As shown in Figure 8A, targeted shRNA against TFEB achieved a significant knockdown of TFEB in cultured cardiomyocytes. Compared to the control group, TFEB knockdown markedly decreased the numbers of autophagosomes and autophagolysosomes after ET-1 challenge, inhibited the increases in myocyte size, and suppressed the hypertrophic gene expression of BNP and β-MHC induced by ET-1. Additionally, TFEB knockdown blunted the myocardial hypertrophy mediated by DGKζ deficiency (Figure 8F-H).

## Discussion

We previously reported that DGKζ inhibits cardiac hypertrophy by suppressing the DAG-PKC pathway. In this study, we discovered a new role for DGKζ in the regulation of Beclin1-mediated autophagy. We observed that DGKζ deficiency resulted in sustained upregulation of Beclin1-mediated autophagy, which contributed to long-term adverse cardiac remodeling and cardiac dysfunction induced by TAC in mice or ET-1 in cardiomyocytes. We demonstrated that DGKζ deficiency prevented the activation of AKT/mTOR signaling, disrupted the association between mTOR and TFEB, and facilitated the nuclear translocation of TFEB from the cytoplasm. These events collectively promoted the activation of Beclin1-mediated autophagy through ULK1/Beclin1 signaling and TFEB-dependent Beclin1 transcription. Genetic inhibition of Beclin1 or TFEB favored the repression of Beclin1-mediated autophagy Vis-a`-Vis suppression of cardiac hypertrophy.

The activation of heterotrimeric Gq protein plays a critical role in cardiac hypertrophy *in vivo* and *in vitro.* Studies have shown that disturbed autophagy in response to GqPCR signaling such as pressure overload, angiotensinⅡmay partially contribute to their prohypertrophic effects 9-12, 15. While the role of autophagy in adverse cardiac hypertrophy remains controversial, Beclin1-dependent autophagy is involved in the pathogenesis of cardiac hypertrophy and heart failure. Beclin1, one of the first identified mammalian autophagy proteins, is a key regulator in autophagy initiation involved in both autophagosome synthesis and maturation. Beclin1 transgenic mice have been reported to display more prominent cardiac remodeling and systolic dysfunction, whereas Beclin-1 haploinsufficiency showed the opposite effect 16. Targeting of beclin1 through activation of transcription factor 3 (ATF3) or miR-30a mimic leads to decreased autophagy and improved pressure overloading or angiotensin II-induced cardiomyocyte hypertrophy 17, 18. Recently, LncRNA AK088388 has been found to regulate beclin1 levels by targeting miR-30a, which further affects autophagy and cell viability in cardiomyocytes 19. In our study, we observed sustained upregulation of the autophagic process, as evidenced by an increase in the LC3Ⅱ and a decrease in the accumulation of p62 in response to hypertrophic stimuli such as pressure overload and ET-1. The upregulated autophagy was further supported by the use of GFP-RFP-LC3 adenovirus. We also found that the increased autophagy was accompanied by a marked upregulation in the expression of Beclin1. Notably, knocking down Beclin1 interrupted autophagy, resulting in the amelioration of cardiac hypertrophy and improvement in left ventricular function. These findings support the involvement of Beclin1-dependent autophagy in long-term pathological remodeling and cardiac dysfunction in the setting of hypertrophic stimuli.

Diacylglycerol kinases (DGKs) consist of a family of enzymes believed to inhibit signaling mediated by GqPCR by reducing the level of DAG *via* conversion of DAG to PA. Currently, ten isozymes of DGK have been discovered in mammals and each subtype may carry out tissue or cell-specific functions. Studies have shown that DGKζ, a predominant isoform in the heart, can prevent cardiac hypertrophy both *in vivo* and *in vitro* 20-22. We previously reported that PPARs suppressed cardiac hypertrophy through inhibition of DAG-PKC pathway in a DGKζ-dependent manner 3, 4. In this study, we extended our results that regulating autophagy may contribute to the ameliorated effect of DGKζ on cardiac hypertrophy. We observed a significant decrease in the level of DGKζ in the hearts of TAC mice and in ET-1-treated cardiomyocytes. The deficiency of DGKζ resulted in enhanced Beclin1-dependent autophagy and the exacerbated cardiac hypertrophy and dysfunction, which could be reversed by silencing Beclin1, indicating the crucial role of DGKζ in regulating Beclin1-dependent autophagy. Furthermore, our findings demonstrated that the knockdown of DGKζ reinforced the inhibition of Akt/mTOR signaling but did not facilitate the activation of AMPK induced by the prohypertrophic signal ET-1. It is well-established that both AKT/mTOR and AMPK play crucial roles in regulating autophagy. AMPK coordinates the induction of autophagy 22. In contrast to AMPK, Akt is activated by phosphorylation and can phosphorylate tuberous sclerosis complex1 (TSC1), which prevents the formation of TSC1-TSC2. This, in turn, allows TSC2 to activate Rheb, leading to the activation of mTOR and the inhibition of autophagy. Conversely, when mTORC1 is inactivated, it separates from ULK1, leading to the dephosphorylation and activation of the ULK1 complex. This, in turn, causes ULK1 to phosphorylate Beclin1 on Ser15, which activates ATG14L-containing VPS34 complexes and triggers the initiation of autophagy 23, 24. Our findings verified that the deletion of DGKζ leads to the decreased phosphorylation of ULK1 but increased phosphorylation of Beclin1 on Ser15, suggesting that DGKζ regulates Beclin1-dependent autophagy through the AKT/mTOR pathway, rather than AMPK signaling.

Several lines of research have indicated that Akt/mTOR signaling is activated in response to hypertrophic stimuli, such as pressure overload, β adrenergic stimulation, angiotensin II and IGF-1, which plays both adaptive and maladaptive roles in the heart 25-27. However, it is important to note that pressure overload-induced inactivation of cardiac Akt/mTOR signaling has also been associated with pathological cardiac hypertrophy and cardiac dysfunction 28, 29. In our study, we observed that the prohypertrophic signal ET-1 induced the inhibition of Akt/mTOR signaling during cardiac hypertrophy, and DGKζ significantly attenuated cardiac hypertrophy by regulating the AKT/mTOR pathway. Previous researches have also demonstrated the involvement of DGKζ in regulating Akt/mTOR pathways in cancer cells, HEK293 cells, and C2C12 myoblasts 30-32. It has been reported that DGKζ-derived PA can directly interact with the mTOR domain and positively regulate mTOR activation 32, 33. Our study found that the deficiency of DGKζ led to a significant increase in DAG levels. Therefore, it is possible that the inhibition of mTOR signaling during cardiac hypertrophy is due to the decreased conversion of DAG to PA caused by DGKζ deletion. However, the exact mechanism by which mTOR activity is regulated after DGKζ deletion requires further clarification.

Our findings suggest that DGKζ can trigger Beclin1-dependent autophagy not only through the activation of Ulk1/Beclin1 signaling but also *via* a TFEB-dependent mechanism. TFEB is a master regulator of autophagy and lysosomal biogenesis, which transcriptionally regulates the expression of various genes involved in the autophagy-lysosome pathway, from cargo recognition and autophagosome formation to vesicle fusion and lysosome-mediated degradation of autophagosomal content. Increasing evidence suggests that upregulation of TFEB leads to an increase in the number of autophagosomes and promotes autophagic flux, while loss of TFEB results in a decrease in the number of autophagosomes and inhibition of lysosome-mediated substrate degradation 34. In normal conditions, TFEB is situated in the cytoplasm. However, during nutrient deprivation or lysosomal stress, TFEB rapidly moves into the nucleus and triggers the expression of downstream genes involved in autophagy and lysosomal biogenesis. Studies have shown that the phosphorylation of TFEB regulates its nuclear translocation and activity, and mTOR has recently been identified as a key regulator of TFEB phosphorylation. Multiple lines of evidence suggest that mTOR-mediated phosphorylation of TFEB keeps it inactive in the cytoplasm. Conversely, inhibition of mTOR dephosphorylates TFEB, leading to its activation and nuclear translocation 35. Consistent with these studies, our findings showed that cardiac hypertrophy induced by TAC *in vivo* and by ET-1 *in vitro* leads to a sustained nuclear accumulation of TFEB. This nuclear accumulation of TFEB is accompanied by a weakened interaction with mTOR, and increased TFEB translocate from the cytoplasm to the nucleus in response to prohypertrophic challenges. Importantly, all of these effects were enhanced by DGKζ deficiency. Furthermore, our results showed that DGKζ deficiency significantly increased the protein and mRNA levels of Beclin1 following exposure to ET-1. However, genetic deletion of TFEB reversed the increased expression of Beclin1 and cardiac autophagy mediated by DGKζ deficiency. Additionally, we identified a transcription factor binding site for Beclin1 within the promoter of Beclin1 and confirmed the positive regulatory effect of TFEB on Beclin1 transcription using chip and luciferase reporter assays. These results suggest that TFEB-mediated upregulation of Beclin1 expression may contribute to the DGKζ deficiency-induced cardiac autophagy. Nevertheless, it is possible that other genes or proteins involved in the autophagy-lysosome pathway also play a role in the activation of autophagy mediated by DGKζ deficiency, given the versatile transcriptional regulation functions mediated by TFEB. Further studies are required to elucidate the precise mechanisms linking DGKζ and TFEB.

The dysregulation of TFEB expression and activity has been implicated in the progression of various diseases, including Parkinson's disease, Alzheimer's disease, Huntington's disease, and RagCS75Y cardiomyopathy 36-39. Recent studies by the Sebastian and Jihoon groups have shown that overexpression of TFEB specifically in cardiomyocytes increases the expression of autophagy genes and renders the heart more susceptible to chronic pressure overload, ultimately leading to heart failure 40. Conversely, knockdown of TFEB attenuates cardiomyocyte death induced by high-dose TAT-Beclin1 41. Consistent with these findings, our study demonstrated that genetic deletion of TFEB reduced cardiac hypertrophy elicited by DGKζ deficiency.

## Conclusion

This study provides novel insights into the role of DGKζ in the regulation of Beclin1-mediated autophagy through the mTOR/TFEB axis. These findings suggest that targeting the autophagy pathway regulated by DGKζ might be a promising therapeutic strategy for cardiac hypertrophy.

## Supplementary Material

Supplementary figure.
