# Supplementary material for: Deficiency of diacylglycerol Kinase ζ promotes Beclin1-mediated autophagy via the mTOR/TFEB signaling pathway: Relevance to maladaptive cardiac hypertrophy
Source: Int J Med Sci. 2024 Jan 1;21(3):439–53. doi: 10.7150/ijms.88134 (PMC10797681; doi:10.7150/ijms.88134)
Supplement: Supplementary file 1 — Supplementary figure. [file ijmsv21p0439s1.pdf]

Deficiency of diacylglycerol Kinaseζ promotes Beclin1-mediated autophagy *via* the mTOR/TFEB signaling pathway: Relevance to maladaptive cardiac hypertrophy

Yumei Liu[1,2*], Han Zhang[3*], Yaxian Lin[4*], Peipei Qian[1], Yuan Yin [5], Ganglin Zou[6], Jinxin Zhang[7], Haining Zhang[1]

[1]Key Laboratory of Molecular Target & Clinical Pharmacology and the State & NMPA Key Laboratory of Respiratory Disease, School of Pharmaceutical Sciences & the Fifth Affiliated Hospital, Guangzhou Medical University, Guangzhou, 511436, P.R. China.

[2] Department of Pharmacology, Jiaying University, Meizhou, 514000, P.R. China.

[3] Department of Stomatology, the First Affiliated Hospital, Sun Yat-sen University, Guangzhou 510080, China

[4] Xiamen Cardiovascular Hospital, Xiamen University, Xiamen, 361005, P.R. China.

[5] Affiliated Guangxi International Zhuang Medical Hospital, Guangxi University of Traditional Chinese Medicine, Guangxi, 530021, P.R. China.

[6] Nanhai Mental Health Center, People's Hospital of Nanhai District, Foshan, 528200, P.R. China.

[7] Department of Medical Statistics, School of Public Health, Sun Yat-sen University, Guangzhou, 510080, China

Supplementary Figure

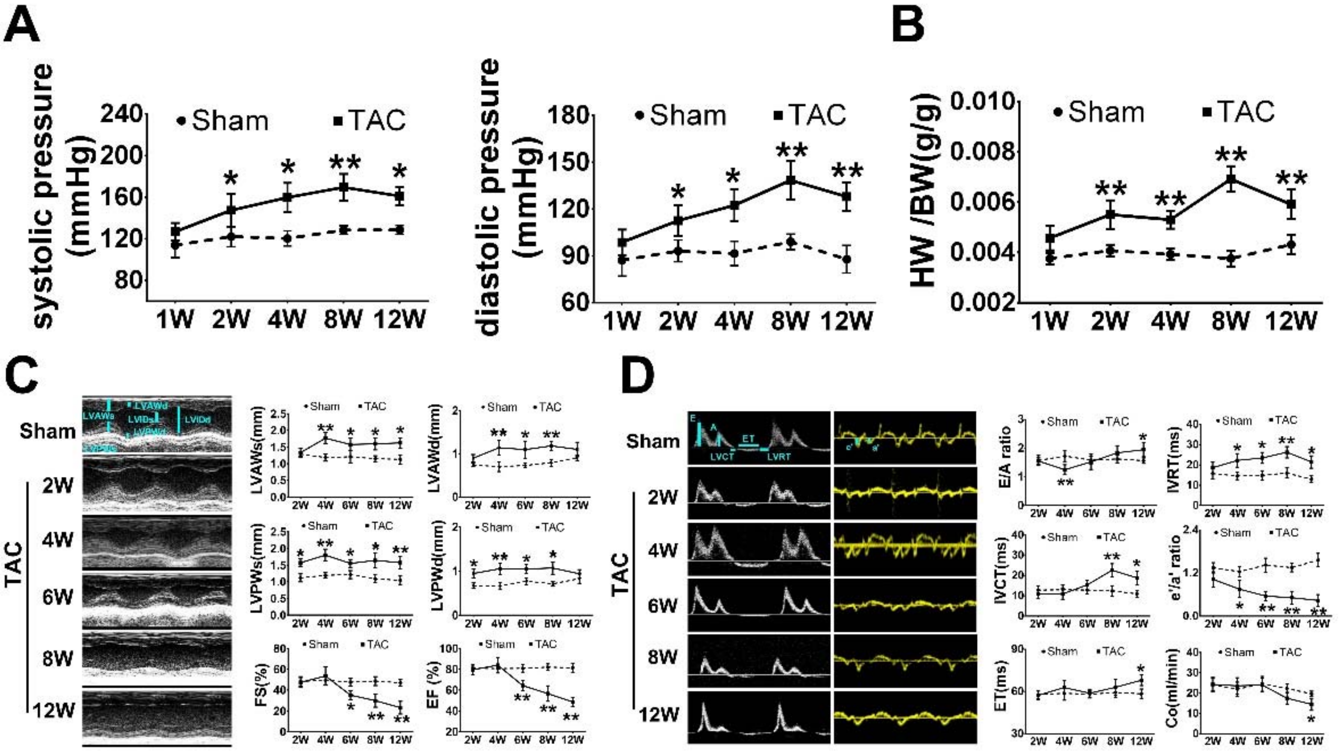

Figure S. Cardiac dysfunction induced by transverse aortic constriction (TAC). Cardiac hypertrophy was induced by ligation of the transverse aortic arch between the innominate artery and the left common carotid artery of mice. (A) Blood pressure was measured by using a small animal non-invasive blood pressure system at indicated time point after surgery (ANOVA for repeated measurements, *n*=6-8, **P*< 0.05 or ***P*< 0.01 *vs.* sham). (B) The ratio of heart weight to body weight (HW/BW) was analyzed at indicated time point after surgery in each experimental group (Student t test, *n*=6-8, ***P*< 0.01 *vs.* sham). (C) Representative M-mode echocardiograms and the analyzed results of cardiac function obtained from mice in each experimental group (ANOVA for repeated measurements, *n*=6-8, **P*< 0.05 or ***P*< 0.01 *vs.* sham). (D) Representative transmitral flow and tissue doppler echocardiograms and the analyzed results of cardiac

function obtained from mice in each experimental group (ANOVA for repeated measurements, n=6-8, *P< 0.05 or **P< 0.01 *vs*. sham).
